# Time to Culture Positivity and Sputum Smear Microscopy during Tuberculosis Therapy

**DOI:** 10.1371/journal.pone.0106075

**Published:** 2014-08-29

**Authors:** Ioana D. Olaru, Jan Heyckendorf, Susanne Grossmann, Christoph Lange

**Affiliations:** 1 Division of Clinical Infectious Diseases, Research Center Borstel, Borstel, Germany; 2 German Center for Infection Research (DZIF), Research Center Borstel, Borstel, Germany; 3 International Health/Infectious Diseases, University of Lübeck, Lübeck, Germany; 4 Department of Medicine, University of Namibia School of Medicine, Windhoek, Namibia; The Catholic University of the Sacred Heart, Rome, Italy

## Abstract

Sputum smear microscopy is widely used for tuberculosis diagnosis and treatment monitoring. We evaluated the correlation between smear microscopy and time to liquid culture positivity during early tuberculosis treatment. The study included patients with smear-positive pulmonary tuberculosis hospitalized at a tuberculosis reference centre in Germany between 01/2012 and 05/2013. Patient records were reviewed and clinical, radiological and microbiological data were analysed. Sputum samples were collected before treatment initiation and weekly thereafter. A number of 310 sputum samples from 30 patients were analysed. Time to liquid culture positivity inversely correlated with smear grade (Spearman's rho −0.439, p<0.001). There was a better correlation within the first two months vs. after two months of therapy (−0.519 vs. −0.416) with a trend to a more rapid increase in time to positivity between baseline and week 2 in patients who culture-converted within the first two months (5.9 days vs. 9.4 days, p = 0.3). In conclusion, the numbers of acid-fast bacilli in sputum smears of patients with pulmonary tuberculosis and time to culture positivity for *M. tuberculosis* cultures from sputum are correlated before and during tuberculosis treatment. A considerable proportion of patients with culture conversion after two months of therapy continued to have detectable acid-fast bacilli on sputum smears.

## Introduction

Tuberculosis (TB) continues to be a leading cause of morbidity and mortality and is currently the fourth most common cause of death from communicable diseases worldwide [1]. According to World Health Organization (WHO), it is estimated that 8.6 million new TB cases occurred in 2011 worldwide resulting in 1.3 million deaths [2]. Although there were major developments in diagnostic methods in recent years with the introduction of automated molecular assays, TB treatment response is still evaluated using traditional microbiological techniques such as smear microscopy and sputum culture. Time to liquid culture positivity can be used as a measure of bacterial burden and has been shown to have an excellent inverse correlation with the number of colony forming units on solid media [3]. Similarly, smear microscopy has been shown to reflect the extent of disease with higher smear grades being associated with a more extensive lung involvement and the presence of cavitary lesions [4]. Smear microscopy provides rapid results, is inexpensive, easy to perform, does not require complex laboratory equipment and is therefore very suitable for low-resource settings. Cultures on the other hand are more expensive and results are available late.

Our objective was to evaluate the correlation between sputum smear microscopy and time to culture positivity during TB treatment.

## Methods

### Study population and setting

Observational cohort study of patients hospitalized with pulmonary TB at the Clinical Tuberculosis Center of the German Center for Infection Research (DZIF), the Medical Clinic Borstel, Germany. Patient records were reviewed and data on clinical, radiological and bacteriological characteristics were collected on all patients with pulmonary TB admitted between January 2012 and May 2013 with at least two positive sputum smear results were further analysed. HIV testing was performed in all patients. Patients received weight-based daily anti-TB therapy according to drug susceptibility testing results and the medical staff observed each drug administration. Patients were hospitalized until acid-fast bacilli became undetectable on sputum smears, or in the case of patients with multidrug-resistant TB, until culture conversion.

### Sample collection and processing

Early-morning sputum specimens were collected before the initiation of anti-TB therapy and weekly thereafter until sputum smear and culture conversion. Samples were processed according to standard bacteriological procedures. Briefly, sputum samples were digested and decontaminated by adding equal volumes of sputum and N-acetyl-L-cysteine – sodium hydroxide solution. After 20 minutes the mixture was neutralized with phosphate buffered saline and centrifuged. The sediment obtained was resuspended in 1 ml sterile phosphate buffer and used for smear preparation and inoculation in culture [5]. Sputum smear grading was assessed using the Ziehl-Neelsen staining method as shown in **Table 1** [6]. Liquid cultures were performed using the BACTEC MGIT 960 system (Becton Dickinson, Sparks, MD). Time to culture positivity was defined as the number of days between sample inoculation and detection of mycobacterial growth. Drug susceptibility testing was performed on all positive initial cultures. Conversion was defined as change from positive to repeatedly negative (three consecutive samples) sputum smear microscopy and negative culture results (culture conversion). Patients were considered to have a negative microscopy after sustained smear conversion (three consecutive negative microscopy results). Sputum smear conversion under effective treatment defines a time point when the infectiousness of patients has become very low. In Germany, patients with drug-susceptible TB are generally discharged from the hospital when sputum smear conversion occurs while on effective treatment, while patients with MDR-TB are generally discharged upon culture conversion, to minimize the risk of transmission of MDR-strains of *M. tuberculosis* in the community [7].

**Table 1:** Quantitative assessment of sputum specimens using microscopy [6].

Result	Number of acid-fast bacilli
	Bright light technique with 100-fold objective magnification
negative	0 on smear (300 fields examined)
±	1–12 on smear (300 fields examined)
+	4–10 in 100 fields (100 fields examined)
++	1–10 in 10 fields (100 fields examined)
+++	1–10 per field of vision (50 fields examined)
++++	>10 per field of vision (20 fields examined)

### Radiological findings

The extent of lung involvement was assessed by two trained physicians using the radiological score described by Ralph et al. [4], which takes into account the proportion of lung affected and the presence of cavitation.

### Statistical analysis

Data processing and analysis were performed using SPSS v17.0 (SPSS Inc., Chicago, IL, USA). Spearman's correlation coefficient was used to assess the strength of associations between continuous and ordinal variables. The Mann-Whitney test was used for comparisons between groups. GraphPad Prism (GraphPad Software Inc. San Diego, CA) statistical software was used to design the figures.

### Ethical approval

The Ethical Board of the University of Lübeck was consulted and it was decided that the anonymous retrospective data analysis did not need ethical board approval. Informed consent was not obtained. Patient data were anonymized and de-identified prior to analysis.

## Results

Of 54 patients diagnosed with TB during the study period, 30 had at least two positive sputum smear results and were included in the final analysis. Patients had a median age of 47.5 years (IQR 39–60) and 23 (77%) were male. According to their country of birth, 16 (53%) were born in Germany, whereas the rest were not German-born. All patients were HIV-seronegative. Seventeen (57%) of patients were smokers and 7 (23%) were alcohol abusers. Diabetes mellitus had been diagnosed in 3 (10%) patients.

### Microbiological characteristics

**Figure 1:**
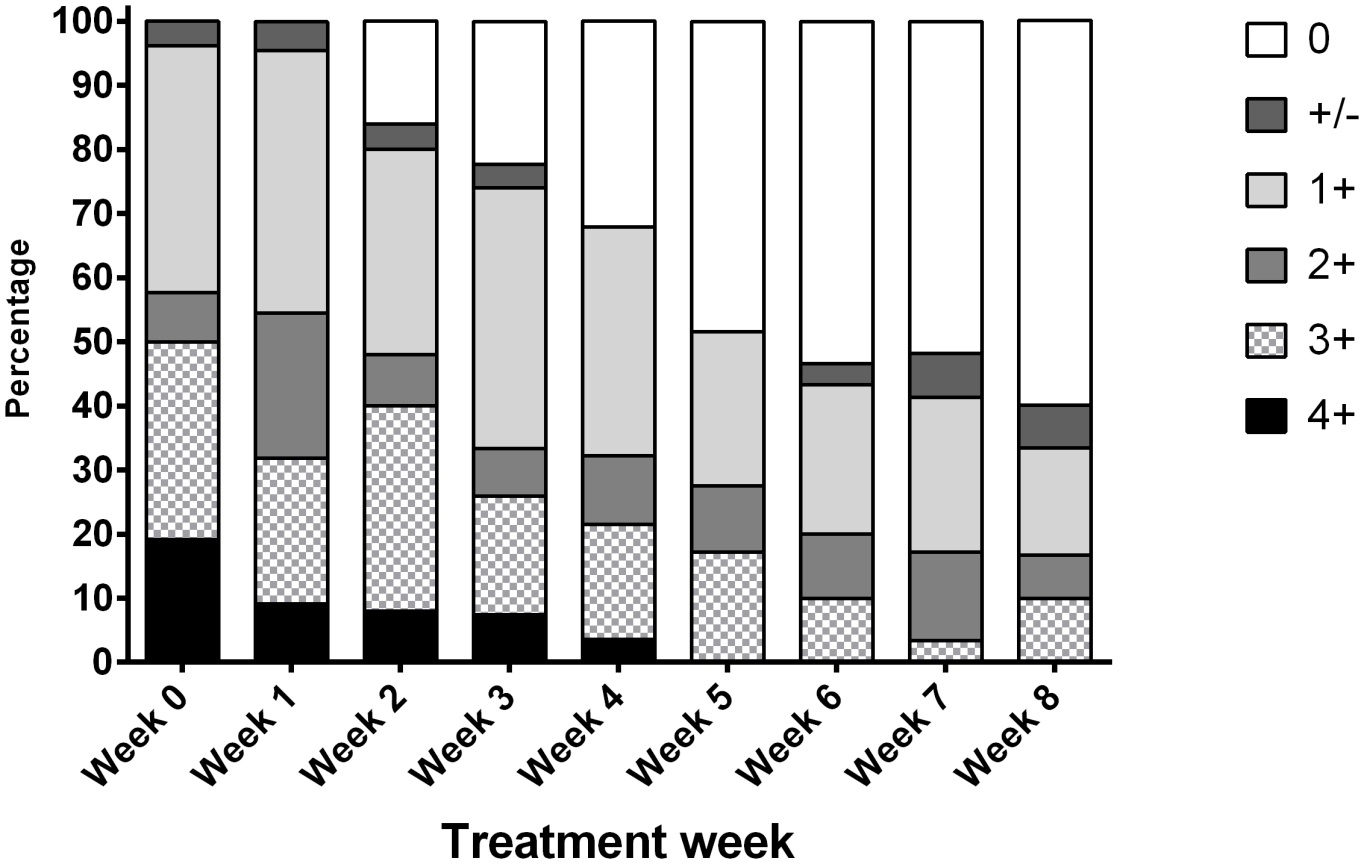
The evolution of smear microscopy grade during the first 8 weeks of tuberculosis treatment.

Drug susceptibility testing (DST) results were available in all patients. Twenty-seven (87%) patients had pan-drug-susceptible TB. One patient had extensively drug-resistant TB, while the other two had isoniazid drug-resistance, one of them in combination with streptomycin drug-resistance. A total of 310 sputum samples were analysed. At treatment initiation, median time to culture positivity was 10.5 (IQR 7–13 days) and increased gradually during the course of treatment. At the start of treatment 13 (50%) of patients had numerous AFB bacilli on microscopy with smear grades of 3+ and 4+. **Figure 1** shows the evolution of smear grade during therapy.

**Figure 2:**
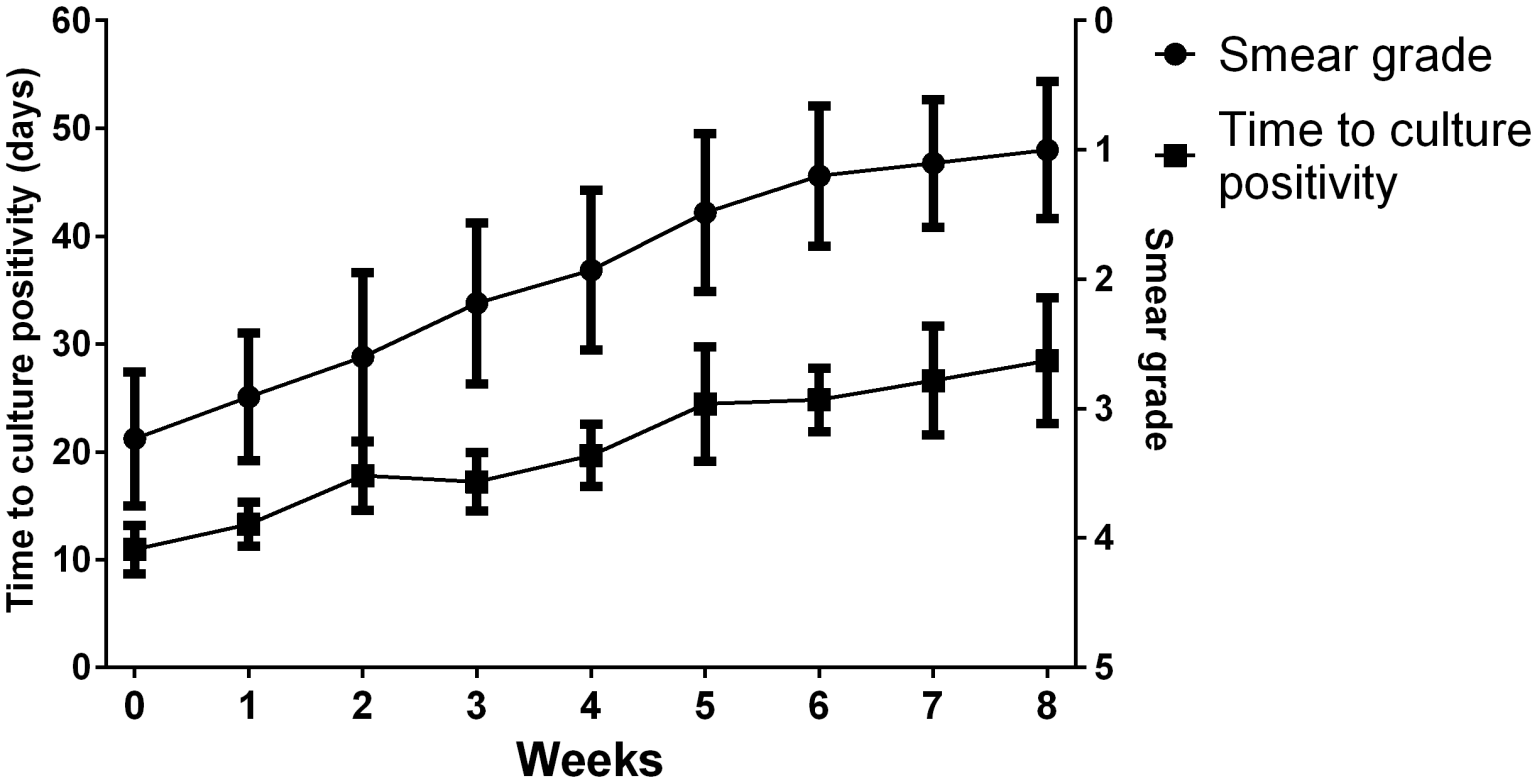
Sputum smear grade and time to liquid culture positivity during tuberculosis therapy (mean values with 95% CI computed using GraphPad Prism are shown). Smear grades are expressed according to the number of visible acid-fast bacilli (AFB) with 0 (no detectable AFB on slide); 1 (1–9 AFB in 300 oil immersion fields), 2 (4–39 AFB in 100 fields), 3 (4–9 AFB in 10 fields), 4 (1–9 AFB per field), 5 (≥10 AFB per field).

There was a negative correlation between smear microscopy and time to culture positivity during the course of treatment (Spearman's correlation coefficient −0.439, p<0.001). The evolution of smear microscopy grade and time to culture positivity during anti-TB treatment is shown in **Figure 2**.

Median time to sputum smear conversion was 60 days (range 20–210 days), while median time to culture conversion was 57 days (range 21–151 days). Fifteen (52%) and 16 (57%) patients underwent smear and culture conversion during the first two months of therapy. Culture conversion occurred between 95 days before smear conversion to 24 days after smear conversion. Six patients (22%) experienced first smear followed by culture conversion, in 9 patients (33%) smear and culture conversion were documented simultaneously from the same specimens, while in 12 patients (45%) smear conversion occurred after culture conversion with a median delay of 22 (range 7–95) days. However in 5 patients (19%) there was a more important delay in smear conversion, which exceeded 1 month. For 3 patients the relationship between the two events could not be precisely determined.

In patients who experienced smear conversion within the first 2 months, culture conversion also occurred in 11 (86%) patients within 2 weeks, while in those who smear-converted after 2 months of therapy, cultures became negative within 14 days in only 5 (36%) patients. There was a significant difference in the delay between culture and smear conversions between patients with a smear conversion before vs. after 2 months of therapy (U = 19, p<0.001).

**Figure 3:**
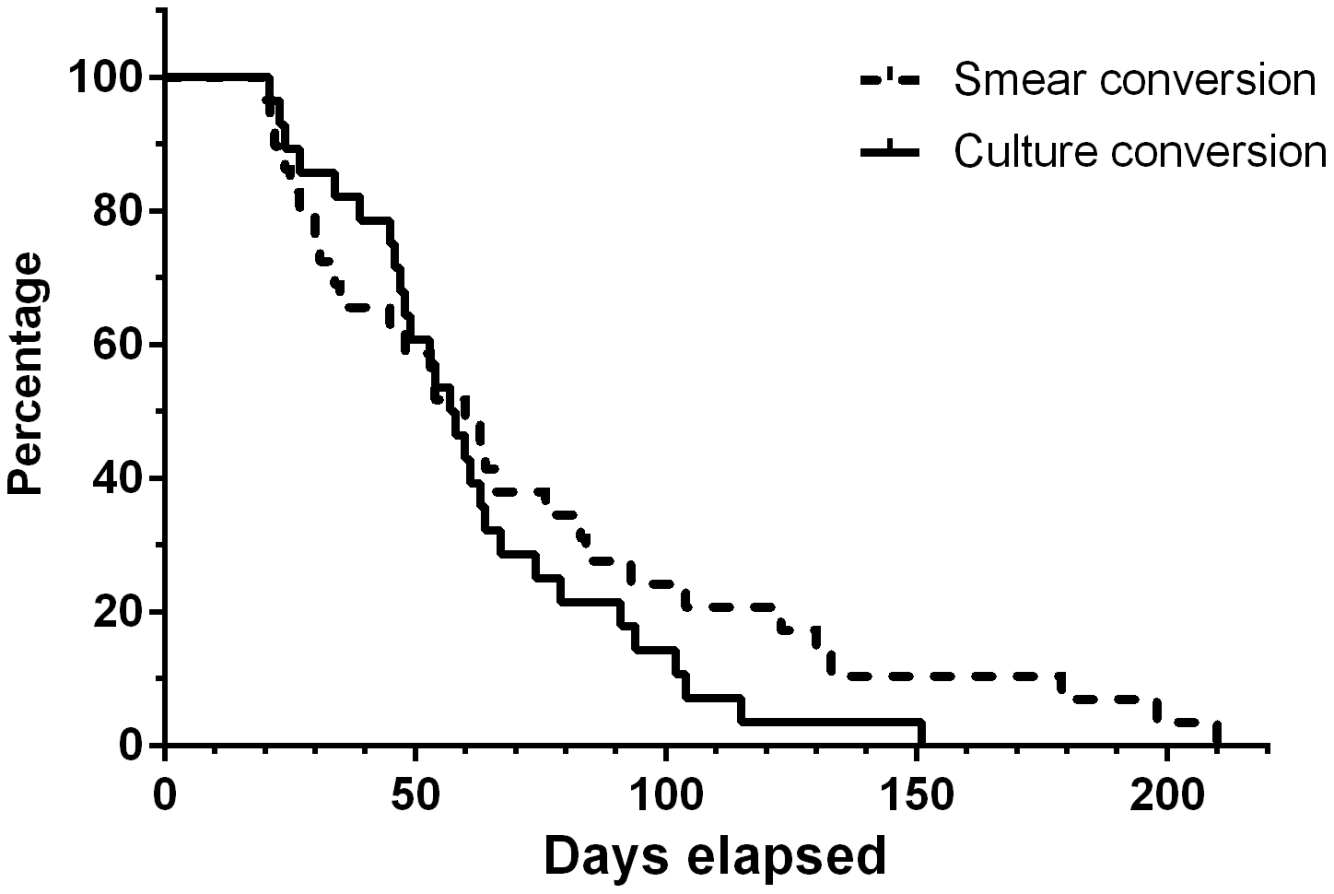
Sputum smear conversion and liquid culture conversion during tuberculosis therapy.

Patients with culture conversion within the first 2 months also had a longer time to culture positivity (U = 23, p = 0.014) and tended to have a lower smear grade at baseline (U = 43, p = 0.08). There was a stronger inverse correlation between smear microscopy and time to culture positivity for patients who experienced smear conversion within the first 2 months (Spearman's rho = −0.519, p<0.001) than for patients who had a delayed smear conversion (Spearman's rho = −0.416, p<0.001). A non-significant higher increase in the difference between time to positivity measured at week 2 and the beginning of treatment was observed in patients who underwent culture conversion before month 2 (U = 31.5, p = 0.3). The mean 11 (IQR 2–15) increase in the number of days was 9.4 days in patients who smear converted vs. 5.9 days 4 (IQR 2–8) in patients who did not. **Figure 3** shows the occurrence of smear and culture conversion during treatment.

### Radiological findings

Chest X-rays were available for assessment for 29 patients. Cavitary lesions were present in 27 (93%) and 19 (66%) had bilateral lung involvement. Median chest X-ray score was

66 points (IQR 52–77.5). The X-ray score did not correlate with baseline smear grade (Spearman's rho = 0.15, p = 0.473) or time to culture positivity (Spearman's rho = 0.284, p = 0.189) and was not associated with sputum smear conversion at 2 months (U = 72, p = 0.355). In the 5 patients who had a time delay between smear and culture conversion longer than 1 month, higher X-ray scores were observed, with a median of 72.5 points (IQR 62.5–83.8) (p = 0.291).

## Discussion

The study assessed the correlation between time to liquid culture positivity and sputum smear grade during the early course of TB therapy. For this purpose serial sputum samples from patients with pulmonary TB were analysed at a clinical TB reference centre in a country of low TB incidence.

Many TB patients come from low-resource settings where laboratories rely on sputum microscopy for diagnosis and treatment monitoring. Although great progress has been made for the rapid diagnosis of TB using molecular techniques such as the Xpert MTB/RIF and there have been extensive efforts of implementing them at concessional prices in developing countries [8], treatment monitoring using molecular assays is difficult due to their inability to differentiate between dead and live bacteria [9]. In a study assessing the performance of Xpert MTB/RIF for monitoring treatment response the test correlated well with sputum smear and culture during therapy. However, after 8 weeks of treatment the proportion of positive Xpert MTB/RIF results was twice that of positive liquid cultures (42 vs. 84%) and an important difference between the two persisted during the remainder of the patient follow-up showing the limitations of this method for treatment monitoring [10].

Sputum culture conversion at 2 months is the only widely accepted marker of sterilizing activity and efficacy and is used for monitoring therapy in patients with pulmonary TB [11], [12]. Liquid culture has become routine microbiology practice and its introduction has improved the sensitivity for detection and reduced the time to result by more than a week in comparison to conventional culture on Löwenstein-Jensen medium [13]. Additionally, liquid culture has a considerably higher sensitivity than smear microscopy. While the limit of detection for culture in liquid media is 10–50 colony forming units/ml, samples must contain about 10,000 colony forming units/ml to be positive on smear microscopy [14]. Nevertheless liquid culture can require more days to weeks until the results are available and therefore cannot be used for rapid decision-making concerning TB-therapy. Time to culture positivity has been shown to correlate to bacterial load as assessed by the number of colony forming units on solid media, and gradually increases during the course of anti-TB treatment [3].

Bactericidal activity studies have shown that bacterial killing does not follow a linear trend. After treatment start there is an initial rapid reduction in the sputum bacterial load that can lead to a 20-fold reduction in the number of colony forming units during the first two days of therapy followed by a further reduction of 200-fold over the next 12 days [15], [16].

In this study a trend to a more rapid increase in time to positivity between baseline and 2 weeks after treatment initiation was observed in patients who underwent culture conversion at 2 months vs. patients who did not. Therefore this measure could possibly be used to identify patients with a better response to therapy. It should be evaluated, whether these patients can benefit from a shorter duration of treatment without an increased risk for relapse. Other authors also found that the rate of increase in time to positivity can predict 2-month smear conversion and thus identify patients with a better overall treatment response [17].

Interestingly, using 2-months sputum smear conversion we could identify two subgroups of patients with different responses to therapy. Patients from the first subgroup underwent smear and culture conversion within the first two months and had a relatively small delay between these two events, whereas patients whose sputum cultures became negative after two months of therapy had more often a longer delay before the occurrence of smear conversion. In another study of patients with smear positive pulmonary TB, it was also noted that up to a third of patients had detectable acid-fast bacilli on sputum smear microscopy and negative *M. tuberculosis* cultures after 2 months of treatment [18]. However, this did not lead to an increased mortality or relapse risk in this patient subgroup [18]. Similarly, persistently positive microscopy results are more likely to be associated with negative cultures, but not with treatment failure and more than 5% of patients have this pattern after 20 weeks of therapy [19]. It has been suggested that this pattern of smear positive, culture negative could be related to rifampicin use, older age and the extent of disease [18], [20]. Persistently positive sputum smears might cause concern and lead to changes in patient management and isolation practices, however this pattern is not uncommon and should not prompt changes in therapy in the absence of signs of disease progression [18]. It has also been shown that about 2% of patients might be smear positive - culture negative at treatment completion and this could be due to non-viable bacteria and colonization by non-tuberculous mycobacteria [21].

In this study, two thirds of patients who underwent culture conversion after 2 months, had AFB present on smear microscopy for more than 2 weeks after cultures had become negative. Although there is a good overall correlation between smear microscopy and time to culture positivity during the first 2 months, the strength of the correlation appears to decrease after 2 months in individual cases. This might be due to the presence of a higher proportion of non-culturable bacteria after this period and suggests that smear grade might be a better predictor of culturable bacteria within the first 2 months of treatment. Other authors have shown that as the smear grade decreases during treatment, the proportion of negative cultures among smear positive patients increases suggesting a rise in the proportion of non-culturable bacteria in the sample [22].

The fact that there are patients who no longer have culturable bacteria in sputum and therefore are likely non-contagious but who continue to have AFB on microscopy stresses the importance of developing new methods that could rapidly and accurately identify this subgroup. A method that might be useful in differentiating between viable and killed mycobacteria is the fluorescein diacetate vital staining. In this method intracellular accumulation of fluorescein occurs in cells with preserved membrane integrity marking cell viability [23]. Fluorescein diacetate vital staining has been used in patients with delayed smear conversion to identify cases of treatment failure earlier [24]. In contrast to sputum smear microscopy and molecular methods culture results are not immediately available. This has an importance because this leads to prolonged hospitalization and therefore increased costs and exposes patients to the risk of infection with drug-resistant TB strains. This underlines the importance of the search for biological markers that are better associated with bacterial killing and sterilizing activity of anti-TB drugs that could be used for monitoring patients under therapy.

The study included only three patients with drug-resistant TB reflecting the low proportion of resistance in the population.

Radiological extent of TB disease evaluated using the proportion of affected lung and the presence of cavities has been shown to be associated to a higher baseline smear grade and can predict smear conversion at 2 months [4]. In this study however radiological score was not associated with smear conversion, possibly due to the small sample size.

This study is limited by the relative small sample size, nevertheless it provides an interesting view on the correlation between the two most important tests used for treatment monitoring of patients with pulmonary tuberculosis and it shows that smear microscopy could provide a rough estimate of the fall in sputum bacterial load particularly during the first two months of therapy. Another limitation of this study is the use of a decontamination procedure of the sputum samples which leads to killing of a fraction of the mycobacteria present. However this a standardized procedure laboratories employed by many laboratories.

In conclusion, there is a strong correlation between time to culture positivity and smear microscopy grade during treatment in patients with pulmonary TB. This finding might be of use to clinicians from resource-limited settings where cultures are difficult to perform or unavailable by supporting the use of smear microscopy in estimating the dynamics of bacterial load during treatment. Two subgroups of patients can be identified: one with conversion within the first two months who had a stronger correlation between time to culture positivity and smear grade and a second group who underwent conversion after two months in whom a greater delay between the two events was present.

## References

[1] Lozano R, Naghavi M, Foreman K, Lim S, Shibuya K, et al (2012) Global and regional mortality from 235 causes of death for 20 age groups in 1990 and 2010: a systematic analysis for the Global Burden of Disease Study 2010. Lancet 380: 2095–2128. 23245604 10.1016/S0140-6736(12)61728-0 PMC10790329

[2] World Health Organization (2013) Global Tuberculosis Report 2013. Geneva, Switzerland.

[3] Bark CM, Okwera A, Joloba ML, Thiel BA, Nakibali JG, et al (2011) Time to detection of Mycobacterium tuberculosis as an alternative to quantitative cultures. Tuberculosis (Edinb) 91: 257–259. 21353641 10.1016/j.tube.2011.01.004 PMC4108903

[4] Ralph AP, Ardian M, Wiguna A, Maguire GP, Becker NG, et al (2010) A simple, valid, numerical score for grading chest x-ray severity in adult smear-positive pulmonary tuberculosis. Thorax 65: 863–869. 20861290 10.1136/thx.2010.136242

[5] Kent PT, Kubica GP (1985) Public health microbiology, a guide for the level III laboratory. Centers for Disease Control, Division of Laboratory Training and Consultation, Atlanta, GA.

[6] (2011) DIN 58943-32 Medical Microbiology - Diagnosis of Tuberculosis - Part 32: Detection of mycobacteria by microscopic methods; Edition 2011-03, Beuth Publishing House.

[7] Lange C, Abubakar I, Alffenaar JW, Bothamley G, Caminero JA, et al (2014) Management of patients with multidrug-resistant/extensively drug-resistant tuberculosis in Europe: a TBNET consensus statement. Eur Respir J 44: 23–63. 24659544 10.1183/09031936.00188313 PMC4076529

[8] http://www.who.int/tb/laboratory/mtbrifrollout Accessed July, 21st 2014.

[9] Thomsen VO, Kok-Jensen A, Buser M, Philippi-Schulz S, Burkardt HJ (1999) Monitoring treatment of patients with pulmonary tuberculosis: can PCR be applied? J Clin Microbiol 37: 3601–3607. 10523560 10.1128/jcm.37.11.3601-3607.1999 PMC85703

[10] Friedrich SO, Rachow A, Saathoff E, Singh K, Mangu CD, et al (2013) Assessment of the sensitivity and specificity of Xpert MTB/RIF assay as an early sputum biomarker of response to tuberculosis treatment. Lancet Respir Med 1: 462–470. 24429244 10.1016/S2213-2600(13)70119-X

[11] Mitchison DA (1993) Assessment of new sterilizing drugs for treating pulmonary tuberculosis by culture at 2 months. Am Rev Respir Dis 147: 1062–1063. 8466107 10.1164/ajrccm/147.4.1062

[12] Perrin FM, Lipman MC, McHugh TD, Gillespie SH (2007) Biomarkers of treatment response in clinical trials of novel antituberculosis agents. Lancet Infect Dis 7: 481–490. 17524807 10.1016/S1473-3099(07)70112-3

[13] Pfyffer GE, Welscher HM, Kissling P, Cieslak C, Casal MJ, et al (1997) Comparison of the Mycobacteria Growth Indicator Tube (MGIT) with radiometric and solid culture for recovery of acid-fast bacilli. J Clin Microbiol 35: 364–368. 9003597 10.1128/jcm.35.2.364-368.1997 PMC229581

[14] Lawn SD, Mwaba P, Bates M, Piatek A, Alexander H, et al (2013) Advances in tuberculosis diagnostics: the Xpert MTB/RIF assay and future prospects for a point-of-care test. Lancet Infect Dis 13: 349–361. 23531388 10.1016/S1473-3099(13)70008-2 PMC4844338

[15] Mitchison DA (1990) Infectivity of patients with pulmonary tuberculosis during chemotherapy. Eur Respir J 3: 385–386. 2114306

[16] Jindani A, Aber VR, Edwards EA, Mitchison DA (1980) The early bactericidal activity of drugs in patients with pulmonary tuberculosis. Am Rev Respir Dis 121: 939–949. 6774638 10.1164/arrd.1980.121.6.939

[17] Carroll NM, Uys P, Hesseling A, Lawrence K, Pheiffer C, et al (2008) Prediction of delayed treatment response in pulmonary tuberculosis: use of time to positivity values of Bactec cultures. Tuberculosis (Edinb) 88: 624–630. 18456556 10.1016/j.tube.2008.03.003

[18] van der Kuyp F, Mahan CS (2012) Prolonged positivity of sputum smears with negative cultures during treatment for pulmonary tuberculosis. Int J Tuberc Lung Dis 16: 1663–1667. 23131266 10.5588/ijtld.12.0238

[19] Al-Moamary MS, Black W, Bessuille E, Elwood RK, Vedal S (1999) The significance of the persistent presence of acid-fast bacilli in sputum smears in pulmonary tuberculosis. Chest 116: 726–731. 10492279 10.1378/chest.116.3.726

[20] Kim TC, Blackman RS, Heatwole KM, Kim T, Rochester DF (1984) Acid-fast bacilli in sputum smears of patients with pulmonary tuberculosis. Prevalence and significance of negative smears pretreatment and positive smears post-treatment. Am Rev Respir Dis 129: 264–268. 6421211

[21] Vidal R, Martin-Casabona N, Juan A, Falgueras T, Miravitlles M (1996) Incidence and significance of acid-fast bacilli in sputum smears at the end of antituberculous treatment. Chest 109: 1562–1565. 8769512 10.1378/chest.109.6.1562

[22] Kim YJ, Lee SM, Park BK, Kim SS, Yi J, et al (2014) Evaluation of propidium monoazide real-time PCR for early detection of viable Mycobacterium tuberculosis in clinical respiratory specimens. Ann Lab Med 34: 203–209. 24790907 10.3343/alm.2014.34.3.203 PMC3999318

[23] Kvach JT, Veras JR (1982) A fluorescent staining procedure for determining the viability of mycobacterial cells. Int J Lepr Other Mycobact Dis 50: 183–192. 6180992

[24] Van Deun A, Maug AK, Hossain A, Gumusboga M, de Jong BC (2012) Fluorescein diacetate vital staining allows earlier diagnosis of rifampicin-resistant tuberculosis. Int J Tuberc Lung Dis 16: 1174–1179. 22747903 10.5588/ijtld.11.0166

